# Poverty alleviation and health services for the poor in China: evidence from national health service surveys in 2013 and 2018

**DOI:** 10.1186/s12939-023-02000-7

**Published:** 2023-10-17

**Authors:** Xiaoyun Liu, Mingyue Li, He Zhu, Qinqin Liu, Xueqin Xie

**Affiliations:** 1 China Center for Health Development Studies, Peking University, Beijing, China; 2 Center of Health Statistics and Information, National Health Commission, Beijing, China

**Keywords:** Poverty reduction, Health care needs, Health care utilization, Medical expenses

## Abstract

**Background:**

China has made intensive efforts to eliminate extreme poverty by 2020. This paper aims to evaluate the changes in health service needs, utilization, and medical expenses for poor people during the poverty alleviation period.

**Methods:**

The study used data from national health services surveys in 2013 and 2018. The poor people were identified and certified by the local government. Health service needs, utilization, medical expenses, and reimbursement rates were analyzed and compared between 2013 and 2018, between the poor and the non-poor groups.

**Results:**

People living in poverty were usually elderly, illiterate, and unemployed. The poor people had a significantly higher two-week morbidity rate and a higher prevalence of chorionic non-communicable diseases than the non-poor group. For both the poor and non-poor, health service needs increased between 2013 and 2018. Accordingly, the poor people had more use of outpatient and inpatient services. The annual inpatient admission rates were 20.8% and 13.1% for the poor and non-poor, respectively, in 2018. The average medical expenses per inpatient admission were much lower for the poor than for the non-poor. Out-of-pocket (OOP) payment share decreased from 41.9% to 2013 to 31.9% in 2018 for the poor, while for the non-poor, the OOP rate was much higher (45.4%) and had no significant changes between the two surveys. The reduction in the OOP share occurred mostly in rural areas.

**Conclusions:**

Poverty alleviation in China may have positive effect in improving poor people’s access to health services, and reducing their financial burden due to illness and health service utilization.

## Introduction

Poverty is a long history social challenge faced by the whole world. In 2017, 9.3% of the world’s population lived below the international poverty line mostly in rural areas, and half were children [1]. The COVID-19 pandemic has pushed 100 million additional people into poverty [2]. Poverty as an extreme form of income inequity in society is correlated with worse health performance [3]. Evidence shows that under-five mortality rates among those living in absolute poverty were five times higher than those in higher-income groups [4]. The long-term poverty is even more harmful to health [5]. The Sustainable Development Goals (SDG) set up a global target to complete the eradication of extreme poverty by 2030.

Poverty and poor health are closely interrelated. Poorer health status often occurs among the lowest income group. On the one hand, poor people tend to have poorer health status due to a lack of resources and limited access to health and social services. On the other hand, people with poor health status are more likely to lose their labor productivity and income, together with higher medical expenses, which often drag the patients’ families into extreme poverty [6].

China is the largest developing country in the world with poverty alleviation as a key social challenge for a long history. The Chinese government has made great efforts in poverty alleviation. The 18th CPC National Congress in 2012 indicated that China’s fight against poverty entered a new era. After 8 years of hard work, China announced the successful elimination of extreme poverty 10 years ahead of the schedule set by the 2030 SDG for poverty alleviation [7]. Nearly 100 million rural residents have got rid of poverty. They enjoy assurances of adequate food and clothing and guarantees of access to compulsory education, basic medical service, and safe housing.

These poverty alleviation activities should have a critical impact on poor people’s utilization of health services and their financial burden due to illness and health service utilization. Yet, there is a lack of scientific evidence at the national level to evaluate the effectiveness of poverty alleviation on health and health care. This paper aims to provide empirical evidence in this regard using data from national health services surveys.

## Methods

This study used data from the national health services survey (NHSS) in 2013 and 2018. National Health Commission organized repeated cross-sectional surveys every five years to investigate the health care needs, demands, and utilization of the Chinese population. The NHSS applied stratified random sampling covering the eastern, central and western rural and urban areas of China. The 2013 survey had 156 sample counties across all 31 provinces, with a total sample size of 273,688. The 2018 survey cover the same 156 counties as in 2013 survey with a total sample size of 256,304. The main purpose of the NHSS is monitor and evaluate the changing health care needs, demands and utilization of Chinese residents. In NHSS, all questions were asked by trained investigators. Interviewees were asked about their status of poverty, including the status of poor households and low-income households identified and certified by the local government using the provincial poverty line and low-income line. The poor households held a card issued by the local government to illustrate their status of poverty and/or low-income. Therefore, this definition of poverty in NHSS covers a wider group of poor population than that identified using the national poverty line.

The dependent variables in the study include health service utilization and health expenses. Health service utilization indicators include outpatient service utilization rate in the past two weeks, inpatient admission rate in the past year, and proportion of of outpatient visit in PHC settings. Health expense indicators include average expenses per inpatient admission and proportion of out-of-pocket payment among total inpatient expenses. The paper starts by describing the demographic characteristics of poor people. It then analyzes and compares the health care needs, health care utilization, and medical expenses between the poor and non-poor. A comparison is also made between the two surveys in 2013 and 2018 to show the effectiveness of poverty alleviation. Multivariate regress analysis was applied to examine the association between poverty status and health service utilization and medical expenses in 2013 and 2018. The differences of coefficients between 2013 and 2018 were examined by Chow test. Average inpatient expenses were transformed into logarithm before fitting linear regression. Factors that may confound health care utilization and inpatient expenses were adjusted, including predisposing characteristics, enabling resources and health care need.

## Results

### Demographic characteristics of the poor

Table 1 shows that being poor was usually correlated with other vulnerability statuses. First, poverty was more prevalent in older adults in rural areas. In 2018, 30.8% of poor and 22.6% of non-poor people were aged over 65 years old in rural areas. Second, the poor were more likely to be illiterate. 24.5% of poor and 11.7% of non-poor people were illiterate in 2018. Third, poverty was associated with unemployment, especially in urban areas. In 2018, 49.1% of the poor and 12.4% of the non-poor were unemployed or out of labor force. However, both the poor and non-poor had high coverage of basic medical insurance schemes (> 98% in 2018).

**Table 1:** Demographic characteristics of the poor between 2013 and 2018 in China

	Total				Urban area				Rural area			
	2013		2018		2013		2018		2013		2018	
	N	%	N	%	N	%	N	%	N	%	N	%
**Aged over 65**												
The poor	7896	34.6	8510	29.9	1753	26.6	768	22.8	6143	37.9	7742	30.8
The non-poor	63,077	28.1	52,664	25.2	29,193	35.3	19,627	30.2	33,884	23.9	33,037	22.9
Total	70,973	28.7	61,174	25.7	30,946	34.6	20,395	29.8	40,027	25.3	40,779	24.1
Chi Square	430.3		291.5		204.0		83.5		1500		735.8	
*P-value*	< 0.001		< 0.001		< 0.001		< 0.001		< 0.001		< 0.001	
**Illiterate**												
The poor	4429	22.5	6159	24.5	540	9.2	337	10.9	3889	28.1	5822	26.4
The non-poor	21,368	11.3	21,085	11.7	3471	4.7	2427	4.2	17,897	15.5	18,658	15.2
Total	25,797	12.3	27,244	13.2	4011	5.0	2764	4.5	21,786	16.8	24,480	16.9
Chi Square	2100		3200		228.9		309.2		1400		1700	
*P-value*	< 0.001		< 0.001		< 0.001		< 0.001		< 0.001		< 0.001	
**Unemployment or out of labor force**												
The poor	6384	32.4	11,603	46.1	2581	43.9	1521	49.1	3803	27.5	10,082	45.7
The non-poor	27,450	14.5	44,928	24.8	9241	12.5	7247	12.4	18,209	15.7	37,681	30.8
Total	33,834	16.2	56,531	27.4	11,822	14.8	8768	14.3	222,012	17.0	47,763	33.0
Chi Square	4200		5000		4200		3200		1200		1900	
*P-value*	< 0.001		< 0.001		< 0.001		< 0.001		< 0.001		< 0.001	
**Covered by health insurance**												
The poor	20,592	96.4	24,758	98.4	5727	91.2	2964	95.6	14,865	98.6	21,794	98.8
The non-poor	199,351	97.0	177,494	98.1	74,029	94.7	57,035	97.6	125,322	98.4	120,459	98.3
Total	219,943	96.9	202,252	98.1	79,756	94.5	59,999	97.5	140,187	98.4	142,253	98.4
Chi Square	18.8		10.9		141.8		51.3		6.9		26.2	
*P-value*	< 0.001		0.001		< 0.001		< 0.001		0.008		< 0.001	

### Health care needs of the poor

Table 2 shows that the poor have higher and increasing health care needs. 45.5% of poor people reported illness in the two weeks prior to the interviews in 2018, a huge increase from 2013 (28.2%), while the non-poor reported a much lower morbidity rate (37.7% in 2018). The prevalence of chronic non-communicable diseases showed a similar changing trend. Poor people reported a higher prevalence (44.2% in 2018) than the non-poor (33.4%), increasing from 31.9% to 2013. The poor also reported poorer health status (averagely scored 68.2 on a 0-100 scale, with 100 indicating perfect health) compared with the non-poor (averagely scored 77.7).

**Table 2:** Health care needs of the poor in 2013 and 2018 in China

	Total		Urban area		Rural area	
	2013	2018	2013	2018	2013	2018
**Two-week morbidity rate**						
The poor	28.2%	45.5%	32.1%	51.6%	26.6%	44.7%
The non-poor	22.8%	37.7%	29.1%	39.5%	19.1%	36.8%
RR	1.23	1.21	1.10	1.31	1.39	1.21
Chi Square	331.0	654.0	26.4	195.1	501.6	564.2
*P-value*	< 0.001	< 0.001	< 0.001	< 0.001	< 0.001	< 0.001
**NCD prevalence**						
The poor	31.9%	44.2%	34.4%	48.8%	30.9%	43.5%
The non-poor	23.9%	33.4%	30.4%	37.0%	20.1%	31.8%
RR	1.34	1.32	1.13	1.32	1.54	1.37
Chi Square	717.3	1300	46.2	190.7	1000	1300
*P-value*	< 0.001	< 0.001	< 0.001	< 0.001	< 0.001	< 0.001
**Self-reported health status**						
The poor	73.5	68.2	73.8	68.0	73.4	68.2
The non-poor	81.1	77.7	80.0	79.0	81.7	77.2
RR	0.91	0.88	0.92	0.86	0.90	0.88
T-value	68.7	82.0	31.2	37.0	63.4	68.7
*P-value*	< 0.001	< 0.001	< 0.001	< 0.001	< 0.001	< 0.001

RR for risk ratio; NCD for noncommunicable chronic disease

### Health care utilization of the poor

Table 3 illustrates health care utilization in poor and non-poor people. In accordance with the increasing health care needs, poor people reported higher outpatient service utilization in 2018 than in 2013, with two-week outpatient utilization rates increasing from 11.5% to 2013 to 18.6% in 2018. The one-year inpatient admission rates increased as well, from 12.9% to 2013 to 20.8% in 2018.

**Table 3:** Health care utilization of the poor in 2013 and 2018 in China

	Total		Urban area			Rural area	
	2013	2018	2013		2018	2013	2018
**Two-week outpatient utilization rate (%)**							
The poor	11.5%	18.6%	10.8%		17.5%	11.8%	18.8%
The non-poor	8.5%	15.9%	8.2%		14.2%	8.7%	16.6%
RR	1.36	1.18	1.32		1.23	1.36	1.13
Chi Square	237.2	139.3	55.6		27.2	174.2	71.2
*P-value*	< 0.001	< 0.001	< 0.001		< 0.001	< 0.001	< 0.001
**One-year inpatient admission rate (%)**							
The poor	12.9%	20.8%	12.8%		17.9%	13.0%	21.2%
The non-poor	9.2%	13.1%	10.3%		13.4%	8.6%	13.0%
RR	1.41	1.59	1.25		1.34	1.52	1.63
Chi Square	337.3	1200	42.2		56.1	347.2	1200
*P-value*	< 0.001	< 0.001	< 0.001		< 0.001	< 0.001	< 0.001
**% of outpatient in PHC settings**							
The poor	73.2%	74.2%	44.5%		49.0%	82.2%	76.9%
The non-poor	60.4%	62.7%	26.3%		32.3%	76.0%	72.6%
RR	1.21	1.18	1.69		1.52	1.08	1.06
Chi Square	146.5	238.6	83.6		57.5	34.5	34.4
*P-value*	< 0.001	< 0.001	< 0.001		< 0.001	< 0.001	< 0.001

RR for risk ratio; PHC for primary health care

A total of 74.2% of the poor group used outpatient services in primary health care (PHC) settings in 2018, which was dramatically higher than that of the non-poor (62.7%). The poor living in rural areas were more likely (76.9%) to use PHC services than their urban counterparts (49.0%).

Multivariate Logistic regression results shows that after controlling potential confounding factors, the poor had higher utilization rate of outpatient services in 2013 (OR = 1.152), but no significant difference in 2018 (OR = 1.008), indicating the growth rate of the poor people’s utilization of outpatient care was lower in comparison to the non-poor. However, for the inpatients service utilization, the OR of poverty increased from 1.194 to 2013 to 1.393 in 2018 (P < 0.01) indicated that the poor people had a higher growth rate of inpatient services utilization than the non-poor (Table 4).

**Table 4:** Multivariate Logistic regression results on health care utilization of the poor

Characteristics	(1) Two-week outpatient utilization		(2) One-year inpatient admission	
	2013	2018	2013	2018
**Poverty (ref = non-poor)**	1.152***	**1.008**	1.194***	1.393***
Predisposing characteristics				
Gender (ref = male)	1.130***	1.114***	1.248***	1.160***
Age (ref = 15–34)				
35–45	1.526***	1.274***	0.746***	0.739***
45–60	2.587***	1.895***	0.617***	0.861***
60+	3.710***	2.265***	1.102**	1.485***
Rurality (ref = urban)	1.260***	1.151***	1.124***	1.115***
Marriage (ref = unmarried and other)	1.060**	1.101***	1.618***	1.472***
Education (ref = high school and above)				
Illiterate	1.037	1.041	1.188***	0.937*
Primary school	1.096***	1.112***	1.345***	1.050*
Junior school	1.004	1.053**	1.179***	1.005
Occupation (ref = employment)				
Unemployment or out of labor force	1.055*	1.142***	1.456***	1.700***
Other	1.273***	1.407***	1.414***	1.302***
Area (ref = eastern)				
Middle	0.667***	0.757***	1.205***	1.214***
Western	0.853***	0.915***	1.321***	1.324***
Enabling resources				
Insurance (ref = not insured)				
Urban employee medical insurance	1.308***	1.167**	2.117***	2.107***
Urban resident medical insurance	1.548***	1.196**	1.539***	1.558***
Urban and rural resident medical insurance	1.382***	1.471***	1.747***	1.684***
New cooperative medical insurance	1.447***	1.218***	1.562***	1.437***
Other	1.693***	1.800***	1.613***	1.860***
Health care need				
Self-rated health (ref = the lowest 1/3 quantile)				
Middle 1/3 quantile	0.444***	0.493***	0.366***	0.410***
The highest 1/3 quantile	0.315***	0.284***	0.270***	0.278***
Observations	209,153	212,616	209,153	212,616

Notes: *** p < 0.001, ** p < 0.01, * p < 0.05. Comparison of poverty coefficient on health care utilization between 2013 and 2018 using Chow test were all significant (P < 0.01)

### Medical expenses and financial burden of the poor

The average inpatient expenses of the poor were less than those of the non-poor. In 2018, the average inpatient expenses were 7776 Yuan and 10,783 Yuan, respectively, with an absolute difference of 3007 Yuan (Table 5).

**Table 5:** Health care expenses of the poor in 2013 and 2018 in China

	Total		Urban areas		Rural areas	
	2013	2018	2013	2018	2013	2018
**Average inpatient expenses (Yuan)**						
The poor	8078 (16,145)	7776 (20,027)	12,021 (23,204)	11,555 (28,356)	6501 (11,877)	7349 (18,811)
The non-poor	9505 (19,080)	10,783 (19,625)	12,486 (22,661)	13,278 (21,620)	7424 (15,788)	9624 (18,512)
t-value	3.9	10.7	0.6	1.8	2.6	7.9
*P-value*	< 0.001	< 0.001	0.565	0.065	0.010	< 0.001
**% of Out-of-Pocket Payment**						
The poor	41.9%	31.9%	44.3%	40.3%	41.0%	31.0%
The non-poor	45.8%	45.4%	40.0%	39.2%	49.8%	48.3%
t-value	3.3	15.0	-1.0	1.3	4.3	15.6
*P-value*	0.001	< 0.001	0.315	0.203	< 0.001	< 0.001

In 2018, the poor had fewer out-of-pocket (OOP) payments than the non-poor, and the percentages for the poor and non-poor were 31.9% and 45.4%, respectively. The percentages of OOP payments decreased dramatically in poor people from 41.9% to 2013 to 31.9% in 2018. The rural (41.0% in 2013 versus 31.0% in 2018) observed a greater decrease in this percentage than the urban areas (44.3% in 2013 versus 40.3% in 2018) among poor people. However, the percentages of OOP payments in 2013 (45.8%) and 2018 (45.4%) were similar among non-poor people .

For both hospitalization expenses and OOP expenses, the OR of poverty decreased from 2013 to 2018, indicating the decreasing financial burden of the poor people over the 5 years (Table 6).

**Table 6:** Multivariate linear regression results on inpatient expenses of the poor

Characteristics	(1) Average inpatient expenses		(2) Out-of-Pocket payment	
	2013	2018	2013	2018
**Poverty (ref = non-poor)**	0.910***	0.800***	0.795***	0.454***
Predisposing characteristics				
Gender (ref = male)	0.813***	0.906***	0.840***	0.942***
Age (ref = 15–34)				
35–45	1.053	1.114***	0.943	0.953
45–60	1.144***	1.102***	0.875***	0.789***
60+	0.948	0.993	0.663***	0.642***
Rurality (ref = urban)	0.859***	0.886***	0.817***	0.843***
Marriage (ref = unmarried and other)	1.037	1.108***	1.072**	1.163***
Education (ref = high school and above)				
Illiterate	0.853***	0.859***	0.848***	0.860***
Primary school	0.862***	0.857***	0.876***	0.879***
Junior school	0.943**	0.949**	0.972	1.027
Occupation (ref = employment)				
Unemployment or out of labor force	1.288***	1.275***	1.339***	1.360***
Other	1.387***	1.301***	1.253***	1.209***
Area (ref = eastern)				
Middle	0.715***	0.710***	0.730***	0.698***
Western	0.709***	0.737***	0.653***	0.658***
Enabling resources				
Insurance (ref = not insured)				
Urban employee medical insurance	1.258***	1.220***	0.601***	0.714***
Urban resident medical insurance	1.120	1.064	0.759***	0.810**
Urban and rural resident medical insurance	1.027	0.837**	0.682***	0.667***
New cooperative medical insurance	0.823**	0.898	0.524***	0.661***
Other	1.131	1.100	0.480***	0.629***
Health care need				
Self-rated health (ref = the lowest 1/3 quantile)				
Middle 1/3 quantile	0.798***	0.905***	0.778***	0.914***
The highest 1/3 quantile	0.696***	0.830***	0.718***	0.850***
Observations	0.798***	0.905***	0.778***	0.914***

Notes: *** p < 0.001, ** p < 0.01, * p < 0.05. Average inpatient expenses and out-of-pocket payment were transformed into logarithm before fitting linear regression. Comparison of poverty coefficient on h medical expenses between 2013 and 2018 using Chow test were all significant (P < 0.01)

## Discussion

This paper analyzed the health care needs, utilization, and medical expenses of poor people in 2013 and 2018 in China. It found that poor people had higher and increasing health care needs than non-poor people. Accordingly, the poor used more health care services. In other words, the poor were not denied access to basic health services because of their poverty status. They had especially higher utilization rate of inpatient services than the non-poor. The poor enjoyed a much higher reimbursement rate than the non-poor, which provided effective financial protection to the poor. Poor people in rural areas benefited more than the urban poor.

The paper reconfirmed that poverty is often correlated with other vulnerability statuses, including illiteracy, aging, and unemployment. All these factors were important social determinants of health. This finding highlights that the focus of poverty alleviation activities should target vulnerable groups with different strategies. It also indicates that the relationship between poverty and health status may be mediated by other social determinants of health. Illiteracy or less education can lead to poorer health status through limited occupational choice and low income, as well as through different thinking and decision-making patterns [7]. Unemployment has a negative impact on health through various mechanisms including the role of relative poverty, social isolation, and health-related behavior [8]. Further research evidence is needed to further clarify the causal relationship between these interlinked social determinants and health in China’s context.

The universal coverage of pension and social health insurance is a key mechanism for the poor to access health services. China started to develop a new cooperative medical scheme in 2003 and a rural pension scheme in 2009 [9]. Almost all rural residents including the poor were enrolled in the two schemes. Evidence has shown that the rural health insurance scheme has made significant contributions to poverty alleviation in rural areas [10].

China’s poverty alleviation movement has made a significant contribution to the financial protection of the poor to use health services. China has regarded development and reform as an important driving force for poverty eradication. Precision poverty alleviation policy requires the precise identification of poor households. For each poverty-stricken household, a specific record or certificate is established to monitor the poverty reduction activities and results. This paper defines the poor as those who had such a record and certificate, although there are many other instruments to measure poverty [11]. One of the key poverty reduction efforts is to provide financial support to the poor to use health services in addition to social health insurance reimbursement [12]. With this precise support, the poor people are able to use the health services they need without further financial hardship. This study found that the OOP payments among the poor group decreased significantly from 2013 to 2018 and were much lower than those among the non-poor group in 2018. The precision poverty alleviation strategy may be the key reason for this achievement.

Although China has achieved the target of poverty elimination by 2020, which has greatly contributed to the poor people’s health service utilization and financial protection, challenges remain to prevent those who have just emerged from poverty from falling into the poverty trap again. The Chinese government promised to guarantee the same protection policy to the poor even after they got rid of poverty. China has started an extensive initiative for rural revitalization. Efforts will be intensified to help those who have emerged from poverty have access to education and employment. These efforts will further guarantee the universal health coverage for the poor and help them create a better life through their own hard work [13].

Guaranteed access to affordable health care does not necessarily mean high quality of care [14]. Further efforts should be made to improve the clinical quality of health services provided to poor people to ensure that the poor group has equal opportunities to use high-quality health care. In addition, the poor group, most of whom are elderly, illiterate, and unemployed may have specific disease patterns, and further studies are needed to investigate the specific health needs of the poor group. Specific clinical services should be organized and provided in response to their health needs.

The study used nationally representative data to analyze the potential impact of poverty alleviation on health care utilization and financial burden. It used two round of national health service survey in 2013 and 2018 to achieve the research objectives. However, this study also has several limitations. First, the two rounds of national health services surveys in 2013 and 2018 occurred among the early and later stages of China’s efforts toward poverty reduction. China only announced the successful completion of poverty elimination by 2020. Later national health services surveys will provide new evidence on the long-term effect of poverty reduction. Second, the before and after comparison cannot distinguish the effect of other social development factors other than poverty reduction.

## Conclusions

The precision poverty reduction strategy has identified the poor people who have a vulnerable socioeconomic status and a higher need for health services. Poverty alleviation efforts may help poor people have equal access to essential health services in accordance with their health needs, and reduce the financial burden of the poor people.

## Data Availability

Data cannot be shared publicly because of confidential concern. Data are available from the Center of Health Statistics and Information of National Health Commission for researchers who meet the criteria for access to confidential data.
